# Impact of myopenia and myosteatosis on postoperative outcome and recurrence in Crohn’s disease

**DOI:** 10.1007/s00384-022-04104-y

**Published:** 2022-02-14

**Authors:** Ioannis Pozios, David Kaufmann, Katharina Boubaris, Hendrik Seeliger, Benjamin Weixler, Andrea Stroux, Carsten Kamphues, Georgios Antonios Margonis, Martin E. Kreis, Katharina Beyer, Claudia Seifarth, Johannes C. Lauscher

**Affiliations:** 1 Department of General and Visceral Surgery, Charité – Universitätsmedizin Berlin, corporate member of Freie Universität Berlin and Humboldt-Universität Zu Berlin, Hindenburgdamm 30, 12203 Berlin, Germany; 2 Department of Diagnostic and Interventional Radiology and Neuroradiology, University Hospital Augsburg, Augsburg, Germany; 3 Charité –Universitätsmedizin Berlin, corporate member of Freie Universität Berlin and Humboldt Universität Zu Berlin, Institute of Biometry and Clinical Epidemiology, Charitéplatz 1, 10117 Berlin, Germany; 4 Berlin Institute of Health at Charité – Universitätsmedizin Berlin, Charitéplatz 1, 10117 Berlin, Germany; 5 Department of Surgery, Memorial Sloan Kettering Cancer Center, New York, NY USA

**Keywords:** Myopenia, Myosteatosis, Crohn’s disease, Anastomotic leakage, Postoperative outcome, Recurrence

## Abstract

**Purpose:**

Myopenia and myosteatosis have been proposed to be prognostic factors of surgical outcomes for various diseases, but their exact role in Crohn’s disease (CD) is unknown. The aim of this study is to evaluate their impact on anastomotic leakage, CD recurrence, and postoperative complications after ileocecal resection in patients with CD.

**Methods:**

A retrospective analysis of CD patients undergoing ileocecal resection at our tertiary referral center was performed. To assess myopenia, skeletal muscle index (skeletal muscle area normalized for body height) was measured using an established image analysis method at third lumbar vertebra level on MRI cross-sectional images. Muscle signal intensity was measured to assess myosteatosis index.

**Results:**

A total of 347 patients were retrospectively analyzed. An adequate abdominal MRI scan within 12 months prior to surgery was available for 223 patients with median follow-up time of 48.8 months (IQR: 20.0–82.9). Anastomotic leakage rate was not associated with myopenia (SMI: *p* = 0.363) or myosteatosis index (*p* = 0.821). Patients with Crohn’s recurrence had a significantly lower SMI (*p* = 0.047) in univariable analysis, but SMI was not an independent factor for recurrent anastomotic stenosis in multivariable analysis (OR 0.951, 95% CI 0.840–1.078; *p* = 0.434). Postoperative complications were not associated with myopenia or myosteatosis.

**Conclusion:**

Based on the largest cohort of its kind with a long follow-up time, we could provide some data that MRI parameters for myopenia and myosteatosis may not be reliable predictors of postoperative outcome or recurrence in patients with Crohn’s disease undergoing ileocecal resection.

## Introduction

Despite the ever-evolving medical therapy for Crohn’s disease (CD), surgery remains a main axis in its therapy, since one in two CD patients require at least one surgical procedure due to complications or refractory symptoms within 10 years after diagnosis [1, 2]. Bowel resection for CD is associated with an increased risk for complications during postoperative course [3–5]. Moreover, after surgery, clinical recurrence rates range from 34 to 86 percent at 3 years [6, 7]. Since high-risk patients could benefit from preventive strategies such as Kono-S anastomosis [8] or postoperative medical prophylaxis [9–11], identifying these patients is essential to improve operative outcomes and reduce recurrence rates and treatment costs.

Multiple factors have been evaluated to predict the postoperative course after surgery for CD [12, 13]. Nutritional status of IBD patients (inflammatory bowel disease) plays a pivotal role in the perioperative management [4], and optimizing nutritional status before surgery reduces postoperative complications and the need for a diverting stoma [14]. However, nutritional status and body contribution are not adequately represented by body mass index (BMI) or serum albumin level [15, 16]. Skeletal muscle wasting and functional compromise occur not only in underweight patients but also in normal-weight and overweight individuals, and serum albumin level is depended on fluid redistribution and inflammation [17, 18]. Thus, novel markers are needed to evaluate surgical patients’ body distribution and nutritional status as potential prognostic factors for postoperative course.

Muscle mass quantity and quality assessment via computed tomography (CT) or magnetic resonance imaging (MRI) represent a highly objective, repeatable, and precise approach for estimation of skeletal muscle depletion [16–18]. Myopenia, defined as the presence of clinically relevant muscle wasting due to any illness and at any age [19], and myosteatosis, the infiltration of fat in skeletal muscle, have been proposed to be prognostic factors of relevant clinical and socio-economic negative outcomes, such as poor surgical outcomes and survival rates for cancer patients [20–23]. However, their exact role in CD is unknown, since only limited data exists showing that myopenia and myosteatosis in CD patients were associated with a higher rate of postoperative complications and unfavorable disease outcome [24, 25]. The aim of the present study was to assess the impact of MRI defined myopenia and myosteatosis on anastomotic leakage after ileocecal resection in patients with CD based on data of a high-volume center. Secondary outcomes were to investigate the role of myopenia and myosteatosis on CD recurrence rates, postoperative complications, and length of hospital stay.

## Methods

### Patients

Patients 18 years or older, who underwent elective ileocecal resection for CD between June 2010 and May 2020 at our tertiary referral IBD (inflammatory bowel disease) surgery center, were retrospectively identified from the hospital’s database. Only patients with available MRI enterography within 12 months before surgery were included in the study. Patients who underwent additional bowel resections were excluded from the analysis. Patients’ demographics, operative outcomes, and CD recurrence rates were collected from the hospital’s electronic health records system. The analysis included age, gender, body mass index (BMI), ASA (American Society of Anesthesiologists) score, presence of vascular disease or preoperative kidney failure, preoperative hemoglobin value, immunosuppressive medication, smoking, surgical techniques, length of surgery, and intraoperative bowel diversion. Rates of anastomotic leakage and reoperation, other postoperative complications such as wound infections, and length of hospital stay were evaluated in order to assess postoperative surgical outcomes. Postoperative complications were graded according to the Clavien-Dindo classification system [26]. Length of hospital stay was defined as the period between primary operation date and discharge from hospital without including readmissions. In order to assess postoperative CD recurrence rates, patients were followed up on a regular basis of 6 months at the Gastroenterology Outpatient Clinic. Postoperative CD recurrence was defined as the reappearance of clinical, radiological, or endoscopic CD manifestations, requiring medical, endoscopic, or surgical treatment.

The study protocol was approved by the Medical Ethical Committee of Charité – Universitätsmedizin Berlin (EA4/148/20).

### Image analysis

**Fig. 1:**
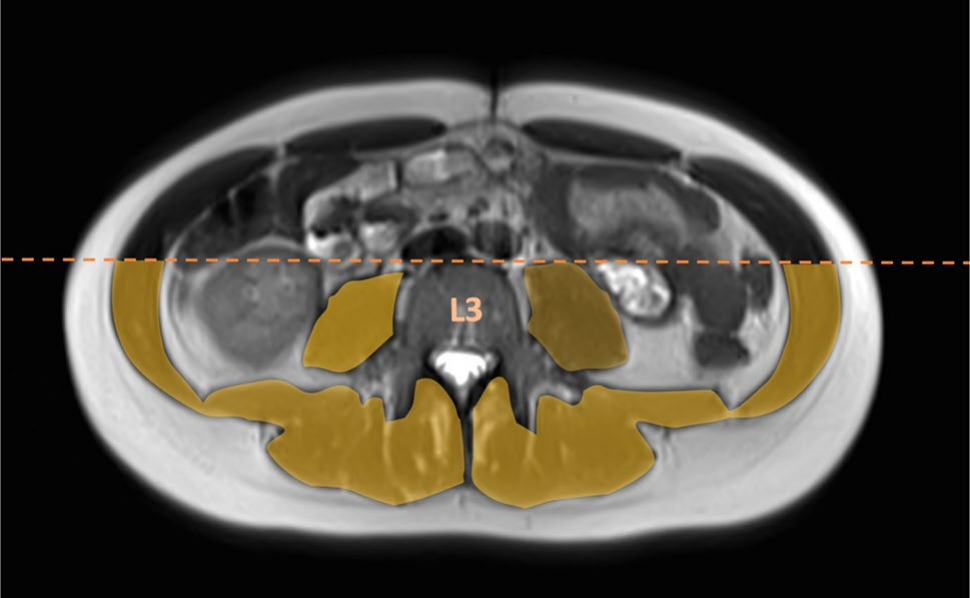
Skeletal muscle area (SMA) on magnetic resonance enterography (MRE). T2-weighted MR images showing the region of interest of dorsal muscles at the level of the third lumbar vertebra (L3)

Image analysis was performed for all patients who underwent MRI at maximum 1 year before surgery including an axial T2 sequence without fat suppression. If a patient had several MRI scans within 1 year before surgery, only the most recent MRI scan was considered for analysis. All MRI scans have been acquired between 2010 and 2020 and were evaluated by a board-certified radiologist (DK) using Visage®7 (Visage Imaging GmbH, Berlin, Germany) software for Microsoft Windows®. Exclusion criteria encompassed poor image quality, large movement or susceptibility artefacts, cut body contours, severe anasarca, and high-grade lumbar spinal stenosis with impaired cerebral spinal fluid (CSF) signal. MRI muscle signal intensity was measured using the approach described by van Dijk et al. [22] in order to assess the presence and extent of myosteatosis. In short, an axial slice at the level of the third lumbar vertebra (L3) with best visualization of both transverse processes was selected. The cross-sectional area of the dorsal skeletal muscles (SMA) including the intermuscular adipose tissue was then determined (cm^2^) at this level as it serves as a representative measure for the total body skeletal muscle mass [27, 28]. The analysis was restricted to the dorsal SMA since the image quality of the ventral section is frequently compromised due to, e.g., movement artefacts. Thereby, the anterior border of the dorsal SMA was set at the level of the anterior edge of L3 (Fig. 1). The average muscle signal intensity of the dorsal SMA was calculated and normalized against the mean cerebrospinal fluid (CSF) signal of the total visible CSF area at the same lumbar level (L3) in order to calculate myosteatosis index. The rationale behind this approach is based on the differences in signal intensity between MR scans and patients, making normalization against an internal standard necessary. Higher (normalized) signal intensity values thereby reflect an increased proportion of fatty tissue, indicating myosteatosis.

In addition, the skeletal muscle index (SMI) was determined by normalizing the dorsal SMA for the squared body height (cm^2^/m^2^), with lower SMI values indicating a reduced muscle mass. The SMI serves as measure for myopenia, which is commonly defined as clinically relevant muscle wasting due to any illness and at any age [19].

### Statistical analysis

Categorical variables were presented as number of patients with corresponding percentages. Quantitative variables were presented as medians with corresponding inter‐quartile ranges (IQRs) for numerical variables. A binary logistic regression power analysis was conducted using the Wald test to determine the sample size. Mann–Whitney U tests and chi-square or Fisher’s exact tests were used for, respectively, continuous and categorical variables to compare independent groups. Fisher’s exact test was used to analyze categorical data when the sample size was small. *p* values lower than 0.05 were considered statistically significant. Univariable analysis were used for our primary endpoint anastomotic leakage and following secondary endpoints: rates of surgical site infections (SSI), reoperation, overall and severe postoperative complications, length of hospital stay, and CD recurrence. Only significant variables from the univariable analysis were entered into the multivariable regression model. Kaplan–Meier curves were performed for recurrence-free rate after surgery, and recurrence-free curves were compared and assessed using the log-rank test. For the determination of low SMI and high muscle signal intensity to define myopenia and myosteatosis, cut-off points were set at the lowest and highest quartile, respectively. Odds ratios (ORs) were calculated with a 95% confidence interval (CI). The statistical analysis was performed using SPSS® Statistics Software 25.0 (IBM®, Armonk, NY, USA).

## Results

### Patients’ characteristics

From June 2010 to May 2020, a total of 347 patients underwent ileocecal resection due to CD at our institution and were retrospectively analyzed. An adequate abdominal MRI scan within 12 months prior to surgery was available for 223 patients, who were finally included in the analysis with a median age of 35 years (IQR: 26–48) and a light female predominance (52%). The median BMI was 21.7 kg/m^2^ (IQR: 19.1–24.5). Forty-six patients (20.6%) were overweight or obese, whereas 43 patients (19.3%) were underweight. Most patients (90.6%) were ASA I or II, and 40.8% of the patients suffered preoperatively from anemia. The majority of the patients (63.2%) were under immunosuppressive therapy, including 5-aminosalicylic acid (5-ASA) (8.5%), steroids (28.3%), azathioprine (21.5%), anti-TNFa (20.6%), or a combination of them. Because of previous abdominal surgery, an open approach was primarily decided in 18.9% of the cases, while in 12.6%, conversion to laparotomy was necessary during the operation. The median operation time was 150 min (IQR: 129–193), and one-quarter of the patients (24.7%) received an ileostomy (Table 1).

**Table 1:** Patients’ characteristics and MRI parameters for myopenia and myosteatosis for CD patients undergone ileocecal resection

**Variable**	*n* = 223
**Preoperative**	
Age, years	35 (26–48)
≤ 35	114 (51.1)
> 35	109 (48.9)
Gender	
Female	116 (52)
Male	107 (48)
BMI, kg/m^2^	21.7 (19.1–24.5)
< 18.5	43 (19.3)
18.5–25	114 (51.1)
≥ 25	46 (20.6)
Myopenia	
SMA, cm^2^	73.1 (57.8–92.2)
SMI, cm^2^/m^2^	25.4 (20.9–29.3)
Myosteatosis	
Muscle signal intensity	0.122 (0.101–0.148)
ASA score	
I–II	202 (90.6)
III–IV	21 (9.4)
Vascular disease	
Yes	30 (13.5)
No	192 (86.1)
Kidney failure	
Yes	23 (10.3)
No	191 (85.7)
Anemia (Hb, g/dl)	
≤ 12	91 (40.8)
> 12	126 (56.5)
Immuno-suppression	141 (63.2)
5-ASA	19 (8.5)
Steroids	63 (28.3)
Azathioprine	48 (21.5)
Anti-TNFa	45 (20.6)
Nicotine	
Yes	28 (16.2)
No	135 (78.0)
Ex-smoker	10 (5.8)
**Intraoperative**	
Surgical approach	
Laparoscopic	152 (68.5)
Conversion	28 (12.6)
Open	42 (18.9)
Operation time, min	150 (129–193)
≤ 150	111 (49.8)
> 150	110 (49.3)
Ileostomy	
Yes	55 (24.7)
No	168 (75.3)

### MRI parameters for myopenia and myosteatosis

Regarding myopenia, median SMA in T2 sequences was 73.1 cm^2^ (IQR: 57.8–92.2). The median SMI was 25.4 cm^2^/m^2^ (IQR: 20.9–29.3). The myosteatosis index had a median of 0.122 (IQR: 0.101–0.148) (Table 1). An analysis of the interval between MRI and surgery time showed no association with myopenia and myosteatosis parameters (data not shown).

### Patient characteristics associated with myopenia and myosteatosis

A significantly lower SMA in MRI scans, an indicator for myopenia, was found in younger patients (median SMA 68.5 vs. 75.5 cm^2^, *p* = 0.022), female patients (59.0 vs. 92.1 cm^2^, *p* < 0.001), patients with a BMI lower than 18.5 kg/m^2^ (58.5 vs. 87.9 cm^2^, *p* < 0.001) or high ASA score (58.5 vs. 74.7 cm^2^, *p* = 0.007), and in patients with preoperative anemia (62.9 vs. 81.6 cm^2^, *p* < 0.001). When SMA was corrected for patients’ body height, i.e., SMI, apart from high ASA score (median SMI 21.3 vs. 25.5 cm^2^/m^2^, *p* = 0.105), young age (23.3 vs. 26.1 cm^2^/m^2^, *p* = 0.016), female gender (21.7 vs. 28.5 cm^2^/m^2^, *p* < 0.001), and low BMI (21.1 vs. 29.7 cm^2^/m^2^, *p* < 0.001) were also significant (Table 2).

**Table 2:** Patients’ characteristics in association with skeletal muscle area, skeletal muscle index, and average muscle signal intensity corrected for cerebrospinal fluid signal intensity in T2-weighted MR images

Variable	Myopenia				Myosteatosis	
	Skeletal muscle area SMA [cm^2^]		Skeletal muscle index SMI [cm^2^/m^2^]		Muscle signal intensity index	
	Median	*p* value	Median	*p* value	Median	*p* value
**Preoperative**						
Age, years						
≤ 35	68.5 (56.4–85.5)	**0.022**	23.3 (20.7–27.9)	**0.016**	0.108 (0.092–0.122)	** < 0.001**
> 35	75.5 (58.6–95.6)		26.1 (21.3–31.3)		0.145 (0.121–0.174)	
Gender						
Female	59.0 (52.4–69.2)	** < 0.001**	21.7 (19.3–25.6)	** < 0.001**	0.129 (0.108–0.160)	**0.001**
Male	92.1 (80.0–103.0)		28.5 (25.5–32.7)		0.114 (0.091–0.143)	
BMI, kg/m^2^						
< 18.5	58.5 (50.8–66.7)	** < 0.001**	21.1 (18.9–23.2)	** < 0.001**	0.110 (0.101–0.137)	**0.007**
18.5–25	74.3 (58.7–93.3)		25.8 (21.4–28.9)		0.121 (0.096–0.143)	
≥ 25	87.9 (71.7–103.2)		29.7 (26.2–33.3)		0.142 (0.113–0.189)	
ASA score						
I–II	74.7 (58.8–93.3)	**0.007**	25.6 (21.1–29.6)	0.105	0.121 (0.100–0.145)	**0.002**
III–IV	58.5 (51.8–73.3)		21.3 (19.6–28.3)		0.178 (0.113–0.209)	
Vascular disease						
Yes	73.7 (55.4–97.8)	0.635	27.6 (21.6–32.5)	0.161	0.170 (0.134–0.211)	** < 0.001**
No	72.2 (58.0–92.0)		24.3 (20.8–29.0)		0.117 (0.099–0.143)	
Kidney failure						
Yes	74.3 (58.0–86.5)	0.840	25.1 (20.8–29.9)	0.848	0.114 (0.101–0.194)	0.431
No	72.3 (57.8–92.1)		25.4 (21.0–29.1)		0.122 (0.100–0.147)	
Anemia (Hb, g/dl)						
≤ 12	62.9 (56.0–82.3)	** < 0.001**	22.6 (20.1–27.0)	** < 0.001**	0.128 (0.108–0.159)	**0.017**
> 12	81.6 (65.2–96.0)		26.9 (22.0–31.4)		0.114 (0.096–0.145)	
Immuno-suppression	70.6 (57.3–95.0)		24.4 (20.7–29.9)		0.121 (0.103–0.146)	
5-ASA	71.3 (50.3–95.0)	0.435	26.0 (18.8–31.9)	0.878	0.130 (0.098–0.177)	0.657
Steroids	70.6 (56.5–95.0)	0.831	23.9 (21.0–30.0)	0.881	0.124 (0.105–0.146)	0.621
Azathioprine	67.9 (56.8–88.7)	0.407	24.1 (20.7–29.3)	0.616	0.125 (0.098–0.143)	0.727
Anti-TNFa	67.8 (55.5–95.4)	0.865	22.2 (20.0–29.0)	0.268	0.114 (0.100–0.147)	0.476
Nicotine						
Yes	71.9 (58.5–96.1)	0.410	24.6 (21.6–30.1)	0.619	0.128 (0.109–0.173)	0.500
No	71.3 (56.9–89.4)		25.0 (21.1–28.5)		0.122 (0.100–0.150)	
Ex-smoker	82.9 (62.6–99.7)		27.6 (20.6–32.7)		0.122 (0.110–0.154)	
**Intraoperative**						
Surgical approach						
Laparoscopic	71.2 (57.8–93.2)	0.513	25.3 (21.3–29.3)	0.540	0.114 (0.100–0.145)	**0.003**
Conversion	70.6 (55.9–86.5)		23.1 (20.2–28.5)		0.131 (0.099–0.146)	
Open	75.8 (58.7–93.4)		26.1 (19.4–31.3)		0.143 (0.121–0.183)	
Operation time, min						
≤ 150	70.6 (55.9–89.7)	0.214	25.5 (21.1–29.7)	0.628	0.126 (0.100–0.154)	0.619
> 150	75.2 (59.0–93.8)		25.2 (20.8–29.1)		0.117 (0.103–0.145)	
Ileostomy						
Yes	66.0 (56.5–85.4)	0.165	23.3 (20.9–28.3)	0.256	0.121 (0.105–0.151)	0.594
No	74.2 (57.8–93.4)		25.9 (20.9–29.9)		0.122 (0.098–0.148)	

Data are described as *n* (%) or median (IQR)

*BMI* body mass index, *ASA* American Society of Anesthesiology, *Hb* hemoglobin, *5-ASA* 5-aminosalicylic acid

Concerning myosteatosis, older patients (median muscle signal intensity index 0.145 vs. 0.108, *p* < 0.001), female (0.129 vs. 0.114, *p* = 0.001), obese or overweight patients (0.142 vs. 0.110, *p* = 0.007), patients with high ASA score (0.178 vs. 0.121, *p* = 0.002), vascular disease (0.170 vs. 0.117, *p* < 0.001), anemia (0.128 vs. 0.114, *p* = 0.017), and patients undergone open surgery (0.143 vs. 0.114, *p* = 0.003) showed significantly higher muscle signal intensity, reflecting higher fat content as indicator for myosteatosis (Table 2).

### Surgical outcomes and recurrence of Crohn’s disease

Overall complications’ rate was 28.7%. Surgical site infections were registered in 28.5% of the patients. Forty-five patients (20.4%) experienced severe postoperative complications (Clavien-Dindo classification IIIa or more), and 44 (19.8%) of them needed redo surgery, with 26 of them (11.7%) having an anastomotic leak. Mortality was zero. The median length of hospital stay was 8 days (IQR: 6–11). The median follow-up time for all patients was 48.8 months (IQR: 20–82.9 months). Overall recurrence of CD affecting the ileocolonic anastomosis was observed in 8.1% of the patients after a median follow-up of 10.2 months (IQR: 5.7–15.4) (Table 3).

**Table 3:** Surgical outcomes and recurrence of Crohn’s disease in association with myopenia and myosteatosis in MR images

**Variable**		**Myopenia**				**Myosteatosis**		
		Skeletal muscle area SMA [cm^2^]		Skeletal muscle index SMI [cm^2^/m^2^]			Muscle signal intensity index	
	*n* = 223	Median	*p* value	Median	*p* value		Median	*p* value
Complication								
Yes	64 (28.7)	69.6 (55.6–94.3)	0.545	24.3 (20.0–29.0)	0.385		0.121 (0.097–0.158)	0.818
No	159 (71.3)	73.7 (58.3–90.0)		25.4 (21.4–29.3)			0.122 (0.102–0.148)	
SSI								
Yes	63 (28.5)	72.3 (55.5–95.0)	0.924	26.0 (19.8–29.3)	0.820		0.120 (0.097–0.158)	0.877
No	158 (71.5)	72.9 (58.4–89.5)		25.2 (21.2–29.1)			0.122 (0.102–0.148)	
Severe complication								
Yes	45 (20.4)	69.1 (55.5–95.4)	0.604	24.1 (19.7–28.2)	0.235		0.120 (0.097–0.164)	0.883
No	176 (79.6)	73.4 (58.5–91.4)		25.5 (21.1–29.5)			0.122 (0.103–0.148)	
Reoperation								
Yes	44 (19.8)	68.4 (55.4–95.5)	0.563	24.0 (19.3–28.5)	0.203		0.120 (0.097–0.166)	0.870
No	178 (80.2)	73.7 (58.5–91.9)		25.6 (21.1–29.6)			0.122 (0.103–0.147)	
Anastomotic leak								
Yes	26 (11.7)	69.3 (55.9–95.0)	0.755	24.3 (20.1–28.0)	0.363		0.120 (0.097–0.176)	0.821
No	196 (88.3)	73.4 (57.9–92.2)		25.4 (21.0–29.5)			0.122 (0.101–0.148)	
LOS, days	8 (6–11)							
LOS ≤ 8	126 (56.5)	72.5 (59.5–93.3)	0.428	25.6 (21.4–29.0)	0.499		0.116 (0.096–0.143)	**0.008**
LOS > 8	96 (43.0)	73.4 (56.6–89.2)		24.3 (20.3–29.4)			0.128 (0.106–0.168)	
Recurrence								
Yes	18 (8.1)	63.0 (57.4–72.9)	0.118	22.5 (20.1–25.4)	**0.047**		0.117 (0.105–0.153)	0.810
No	204 (91.9)	73.9 (57.8–93.2)		25.7 (21.1–29.6)			0.122 (0.100–0.148)	

Data are described as *n* (%) or median (IQR)

*SSI* surgical site infections; Severe complication, grade IIIa-V nach Clavien-Dindo; *LOS* length of hospital stay

### Myopenia and myosteatosis in association with anastomotic leakage after ileocecal resection for Crohn’s disease

To assess in detail our primary endpoint, myopenia and myosteatosis values were evaluated in association with anastomotic leakage rates. Concerning myopenia, neither SMA [median SMA 69.3 cm^2^ (leakage group) vs. 73.4 cm^2^ (*p* = 0.755)] nor SMI [median SMI 24.3 cm^2^/m^2^ (leakage group) vs. 25.4 cm^2^/m^2^ (*p* = 0.363)] was associated with anastomotic leakage after surgery for terminal ileitis (Table 3). In addition, we found no association between myosteatosis index and anastomotic leakage after ileocecal resection [median 0.120 (leakage group) vs. 0.122 (*p* = 0.821)] (Table 3).

### Myopenia and myosteatosis in association with recurrence of anastomotic stenosis

Myopenia and myosteatosis values were evaluated in relation to recurrent stenosis of ileocecal anastomosis. Although SMA was not associated with incidence of CD recurrence [median SMA 63.0 cm^2^ (patients with recurrence) vs. 73.9 cm^2^ (*p* = 0.118)], height adjusted SMA (SMI) was significantly lower in patients with recurrent anastomotic stenosis in univariable analysis [median SMI 22.5 cm^2^/m^2^ (patients with recurrence) vs. 25.7 cm^2^/m^2^ (*p* = 0.047)]. Myosteatosis index was not associated with recurrence rates of anastomotic stenosis in univariable analysis [median 0.117 (recurrence group) vs. 0.122 (*p* = 0.821)] (Table 3).

### Analysis of risk factors for recurrent anastomotic stenosis after ileocecal resection for CD

To identify independent risk factors for CD recurrence after surgery, a multivariable logistic regression was performed. Variables that showed significant association with CD recurrence in the univariable analysis, i.e., sex (*p* = 0.045), myopenia (SMI) (*p* = 0.047), kidney failure (*p* = 0.011), and the presence of ileostomy (*p* = 0.028), were enrolled in the multivariable model. Multivariable analysis identified kidney failure as an independent risk factor for recurrent anastomotic stenosis after ileocecal resection (OR 5.656, 95% CI 1.611–19.852; *p* = 0.007). Although myopenia index (SMI) was significantly associated with a higher incidence of CD recurrence in univariable analysis, in multivariable analysis, SMI was not an independent factor for anastomotic stenosis (OR 0.951, 95% CI 0.840–1.078; *p* = 0.434). Results of univariable analysis and multivariable logistic regression for CD recurrence are shown in Table 4.

**Table 4:** Analysis of risk factors for recurrence of Crohn’s disease after ileocecal resection

**Variable**	**Univariate analysis**			**Multivariable analysis**		
	No recurrence	Recurrence	*p* value	OR	95% CI	*p* value
Age, years						
≤ 35	103 (90.4)	11 (9.6)	0.317			
> 35	101 (94.4)	6 (5.6)				
Sex						
Female	103 (88.8)	13 (11.2)	**0.045**			0.065
Male	101 (96.2)	4 (3.8)		0.310	0.089–1.075	
BMI kg/m^2^						
< 18.5	40 (93.0)	3 (7.0)	0.657			
18.5–25	104 (91.2)	10 (8.8)				
≥ 25	42 (95.5)	2 (4.5)				
Myopenia						
SMA cm^2^	73.9 (57.8–93.2)	63.0 (57.4–72.9)	0.118			
SMI cm^2^/m^2^	25.7 (21.1–29.6)	22.5 (20.1–25.4)	**0.047**	0.951	0.840–1.078	0.434
Myosteatosis						
Muscle signal intensity	0.122 (0.100–0.148)	0.117 (0.105–0.153)	0.810			
ASA score						
I–II	185 (92.5)	15 (7.5)	0.741			
III–IV	19 (90.5)	2 (9.5)				
Vascular disease						
Yes	177 (92.7)	14 (7.3)	0.710			
No	27 (90.0)	3 (10.0)				
Kidney failure						
Yes	17 (77.3)	5 (22.7)	**0.011**	5.656	1.611–19.852	**0.007**
No	181 (94.8)	10 (5.2)				
Anemia (Hb, g/dl)						
≤ 12	82 (91.1)	8 (8.9)	0.493			
> 12	117 (93.6)	8 (6.4)				
Immunosuppression	129 (92.1)	11 (7.9)	0.986			
5-ASA	19 (100.0)	0 (0.0)	0.373			
Steroids	55 (87.3)	8 (12.7)	0.091			
Azathioprine	44 (91.7)	4 (8.3)	0.893			
Anti-TNFa	43 (97.7)	1 (2.3)	0.206			
Nicotine						
Yes	23 (82.1)	5 (17.9)	0.089			
No	125 (93.3)	9 (6.7)				
Ex-smoker	8 (80.0)	2 (20.0)				
Surgical approach						
Laparoscopic	139 (91.4)	13 (8.6)	0.852			
Conversion	25 (96.2)	1 (3.8)				
Open	38 (92.7)	3 (7.3)				
Operation time, min						
≤ 150	102 (91.9)	9 (8.1)	0.831			
> 150	101 (92.7)	8 (7.3)				
Ileostomy						
Yes	47 (85.5)	8 (14.5)	**0.028**	3.029	0.979–9.370	0.054
No	157 (94.6)	9 (5.4)				
Severe complication						
Yes	40 (90.9)	4 (9.1)	0.752			
No	163 (92.6)	13 (7.4)				

Data are described as *n* (%); *OR* odds ratio, *CI* confidence interval, *BMI* body mass index, *SMA* skeletal muscle area, *SMI* skeletal muscle index, *ASA* American Society of Anesthesiology, *Hb* hemoglobin; Severe complication, grade IIIa-V nach Clavien-Dindo

### Recurrence-free survival independent of preoperative myopenia and myosteatosis

**Fig. 2:**
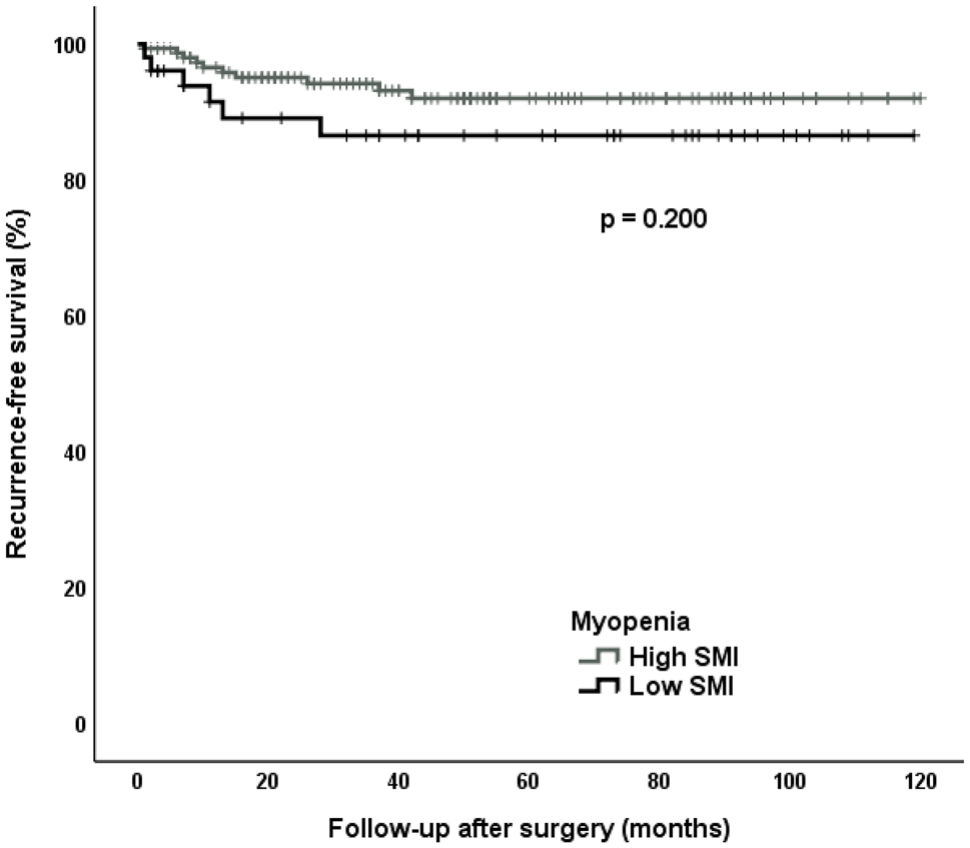
Myopenia effect on recurrent Crohn stenosis. Recurrence-free survival rate after surgery in patients with low and high SMI

**Fig. 3:**
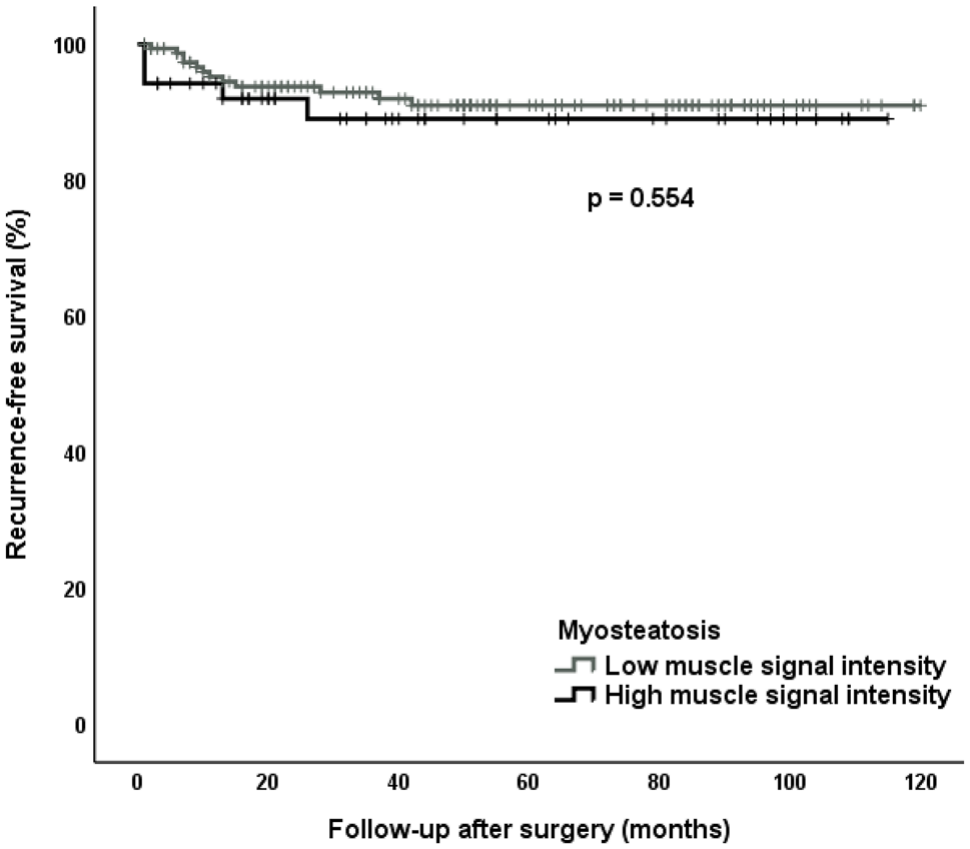
Myosteatosis effect on recurrent Crohn stenosis. Recurrence-free survival rate after surgery in patients with high and low myosteatosis index

In recurrence-free survival analysis, the cut-off value for the lowest quartile for SMI was 20.9 cm^2^/m^2^. In total, a SMI value below the cut-off was present in 51 patients (25%) with 6 patients developing a recurrent Crohn stenosis (11.8%). The median recurrence-free survival time in the low SMI group was 50.0 months (IQR 11.0–89.0) while in the high SMI group was 45.0 months (IQR 20.0–76.0). In the Kaplan–Meier analysis, there were no significant differences in recurrence-free survival between the myopenia group (low SMI) versus the group with high SMI (*p* = 0.200) (Fig. 2). The cut-off value for the highest quartile for skeletal muscle signal intensity (myosteatosis index) was 0.148. A myosteatosis index above the cut-off was present in 52 patients (25%) with 5 patients developing a recurrent Crohn stenosis (9.6%). The median recurrence-free survival time in the patients’ group with low and high myosteatosis index was 50.0 months (IQR 22.5–83.0) and 36.5 months (IQR 16.0–80.5), respectively. In the Kaplan–Meier analysis, this difference was not significant (*p* = 0.554) (Fig. 3).

### Postoperative complications and length of hospital stay in association with myopenia and myosteatosis

No association was found between MRI-defined myopenia values (SMA or SMI) and overall postoperative complications [median SMI: 24.3 cm^2^/m^2^ (complications group) vs. 25.4 cm^2^/m^2^ (*p* = 0.385)], surgical site infections [median SMI: 26.0 cm^2^/m^2^ (SSI group) vs. 25.2 cm^2^/m^2^ (*p* = 0.820)], severe postoperative complications [median SMI: 24.1 cm^2^/m^2^ (severe complications group) vs. 25.5 cm^2^/m^2^ (*p* = 0.235)], reoperation rates [median SMI: 24.0 cm^2^/m^2^ (reoperation group) vs. 25.6 cm^2^/m^2^ (*p* = 0.203)], and length of hospital stay [median SMI: 25.6 cm^2^/m^2^ (LOS ≤ 8) vs. 24.3 cm^2^/m^2^ (LOS > 8); (*p* = 0.499)] in CD patients after ileocecal resection (Table 3). Myosteatosis also did not significantly associate with overall complications’ rate [median 0.121 (complications group) vs. 0.122 (*p* = 0.818)], surgical site infections [median 0.120 (SSI group) vs. 0.122 (*p* = 0.877)], severe postoperative complications [median 0.120 (severe complications group) vs. 0.122 (*p* = 0.883)], or reoperations [median 0.120 (reoperation group) vs. 0.122 (*p* = 0.870)]. However, patients with a prolonged hospital stay (> 8 days) were characterized by a higher signal intensity index, i.e., myosteatosis, in univariable analysis [median 0.128 (LOS > 8) vs. 0.116 (*p* = 0.008)] (Table 3).

### Analysis of risk factors for postoperative complications after ileocecal resection for CD

To identify independent risk factors for postoperative complications after ileocecal resection, a multivariable logistic regression was performed. Variables that showed significant association with postoperative complications in the univariable analysis, i.e., preoperative medication with azathioprine (*p* = 0.033), anti-TNFa (*p* = 0.031), as well as myopenia (SMI) (*p* = 0.385) and myosteatosis (*p* = 0.818), were enrolled in the multivariable model. Multivariable analysis identified no independent risk factors for occurrence of postoperative complications after ileocecal resection in our cohort. Results of univariable analysis and multivariable logistic regression for postoperative complications are shown in Table 5.

**Table 5:** Analysis of risk factors for postoperative complications after ileocecal resection for Crohn’s disease

**Variable**	**Univariate analysis**			**Multivariable analysis**		
	No complication	Complication	*p* value	OR	95% CI	*p* value
Age, years						
≤ 35	83 (72.8)	31 (27.2)	0.234			
> 35	70 (65.4)	37 (34.6)				
Sex						
Female	80 (69.0)	36 (31.0)	0.928			
Male	73 (69.5)	32 (30.5)				
BMI, kg/m^2^						
< 18.5	27 (62.8)	16 (37.2)	0.267			
18.5–25	83 (72.8)	31 (27.2)				
≥ 25	27 (61.4)	17 (38.6)				
Myopenia						
SMA, cm^2^	73.7 (58.3–90.0)	69.6 (55.6–94.3)	0.545			
SMI, cm^2^/m^2^	25.4 (21.4–29.3)	24.3 (20.0–29.0)	0.385	0.999	0.949–1.052	0.971
Myosteatosis						
Muscle signal intensity	0.122 (0.102–0.148)	0.121 (0.097–0.158)	0.818	5.035	0.021–1186.952	0.133
ASA score						
I–II	140 (70.0)	60 (30.0)	0.444			
III–IV	13 (61.9)	8 (38.1)				
Vascular disease						
Yes	21 (70.0)	9 (30.0)	0.922			
No	132 (69.1)	59 (30.9)				
Kidney failure						
Yes	14 (60.9)	9 (39.1)	0.401			
No	132 (69.5)	58 (30.5)				
Anemia (Hb, g/dl)						
≤ 12	64 (71.1)	26 (28.9)	0.626			
> 12	85 (68.0)	40 (32.0)				
Immunosuppression	97 (68.8)	44 (31.2)	0.996			
5-ASA	13 (68.4)	6 (31.6)	0.981			
Steroids	46 (73.0)	17 (27.0)	0.377			
Azathioprine	39 (81.3)	9 (18.8)	**0.033**	0.531	0.233–1.213	0.133
Anti-TNFa	25 (55.6)	20 (44.4)	**0.031**	1.805	0.881–3.698	0.106
Nicotine						
Yes	20 (71.4)	8 (28.6)	0.545			
No	88 (65.2)	47 (34.8)				
Ex-smoker	8 (80.0)	2 (20.0)				
Surgical approach						
Laparoscopic	105 (69.1)	47 (30.9)	0.915			
Conversion	19 (70.4)	8 (29.6)				
Open	27 (67.5)	13 (32.5)				
Operation time, min						
≤ 150	80 (72.1)	31 (27.9)	0.411			
> 150	73 (67.0)	36 (33.0)				
Ileostomy						
Yes	36 (66.7)	18 (33.3)	0.639			
No	117 (70.1)	50 (29.9)				

Data are described as *n* (%); *OR* odds ratio, *CI* confidence interval, *BMI* body mass index, *SMA* skeletal muscle area, *SMI* skeletal muscle index, *ASA* American Society of Anesthesiology, *Hb* hemoglobin

### Analysis of risk factors for prolonged hospital stay after ileocecal resection for CD

To further analyze the association between myosteatosis and length of hospital stay, a multivariable logistic regression was performed. Variables that showed significant association with length of hospital stay in the univariable analysis were enrolled in the multivariable model. Thus, age (*p* < 0.001), myosteatosis (*p* = 0.008), kidney failure (*p* < 0.001), immunosuppression with 5-ASA (*p* = 0.016), surgical approach (*p* = 0.001), presence of ileostomy (*p* = 0.024), and severe complications (*p* = 0.010) are included in the logistic regression model. Multivariable analysis identified five independent risk factors for longer hospital stay after ileocecal resection, including age (OR 2.767, 95% CI 1.446–5.293; *p* = 0.002), kidney failure (OR 4.680, 95% CI 1.376–15.915; *p* = 0.013), surgical approach (OR 2.083, 95% CI 1.035–4.190; *p* = 0.040), presence of ileostomy (OR 2.806, 95% CI 1.319–5.969; *p* = 0.007), and severe complications (OR 2.701, 95% CI 1.248–5.847; *p* = 0.012). Myosteatosis, though, was not an independent factor for prolonged hospital stay (OR 32.118, 95% CI 0.035–29,855.9; *p* = 0.320). Results of univariable analysis for length of hospital stay and multivariable logistic regression are shown in Table 6.

**Table 6:** Analysis of risk factors for length of hospital stay after ileocecal resection for Crohn’s disease

**Variable**	**Univariate analysis**			**Multivariable analysis**		
	LOS ≤ 8 days	LOS > 8 days	*p* value	OR	95% CI	*p* value
Age, years						
≤ 35	79 (69.3)	35 (30.7)	** < 0.001**			
> 35	47 (43.5)	61 (56.5)		2.767	1.446–5.293	**0.002**
Sex						
Female	68 (58.6)	48 (41.4)	0.558			
Male	58 (54.7)	48 (45.3)				
BMI, kg/m^2^						
< 18.5	22 (51.2)	21 (48.8)	0.410			
18.5–25	69 (60.5)	45 (39.5)				
≥ 25	23 (51.1)	22 (48.9)				
Myopenia						
SMA, cm^2^	72.5 (59.5–93.3)	73.4 (56.6–89.2)	0.428			
SMI, cm^2^/m^2^	25.6 (21.4–29.0)	24.3 (20.3–29.4)	0.499			
Myosteatosis						
Muscle signal intensity	0.117 (0.096–0.143)	0.128 (0.106–0.168)	**0.008**	32.118	0.035–29,855.866	0.320
ASA score						
I–II	118 (58.7)	83 (41.3)	0.070			
III–IV	8 (38.1)	13 (61.9)				
Vascular disease						
Yes	13 (43.3)	17 (56.7)	0.111			
No	113 (58.9)	79 (41.1)				
Kidney failure						
Yes	5 (21.7)	18 (78.3)	** < 0.001**	4.680	1.376–15.915	**0.013**
No	115 (60.2)	76 (39.8)				
Anemia (Hb, g/dl)						
≤ 12	53 (58.2)	38 (41.8)	0.832			
> 12	71 (56.8)	54 (43.2)				
Immunosuppression	81 (57.4)	60 (42.6)	0.965			
5-ASA	6 (31.6)	13 (68.4)	**0.016**	0.032	1.111–11.147	3.519
Steroids	34 (54.0)	29 (46.0)	0.546			
Azathioprine	32 (66.7)	16 (33.3)	0.131			
Anti-TNFa	24 (53.3)	21 (46.7)	0.613			
Nicotine						
Yes	15 (53.6)	13 (46.4)	0.275			
No	73 (54.1)	62 (45.9)				
Ex-smoker	8 (80.0)	2 (20.0)				
Surgical approach						
Laparoscopic	97 (63.8)	55 (36.2)	**0.001**			
Conversion	16 (59.3)	11 (40.7)				
Open	12 (29.3)	29 (70.7)		2.083	1.035–4.190	**0.040**
Operation time, min						
≤ 150	66 (59.5)	45 (40.5)	0.383			
> 150	59 (53.6)	51 (46.4)				
Ileostomy						
Yes	24 (43.6)	31 (56.4)	**0.024**	2.806	1.319–5.969	**0.007**
No	102 (61.1)	65 (38.9)				
Severe complication						
Yes	18 (40.0)	27 (60.0)	**0.010**	2.701	1.248–5.847	**0.012**
No	108 (61.4)	68 (38.6)				

Data are described as *n* (%); *LOS* length of stay, *OR* odds ratio, *CI* confidence interval, *BMI* body mass index, *SMA* skeletal muscle area, *SMI* skeletal muscle index, *ASA* American Society of Anesthesiology, *Hb* hemoglobin; Severe complication, grade IIIa-V nach Clavien-Dindo

## Discussion

Our present study with data from a single high-volume IBD center evaluates the role of MRI based assessment of myopenia and myosteatosis on postoperative course and disease recurrence in patients with Crohn’s disease (CD) after ileocecal resection. Our study demonstrated that measurement of the skeletal muscle area and intensity using conventional T2-weighted MRI enterography sequences obtained preoperatively for CD staging was easily feasible. Myopenia was observed in younger, female, underweight patients with preoperative anemia, while myosteatosis in older, female, overweight patients, with high ASA score, vascular disease, and anemia. In our present study, none of the radiological variables for myopenia or myosteatosis was associated with anastomotic leakage, postoperative complications, or CD recurrence.

There are only limited data on the effects of myopenia and myosteatosis on postoperative outcomes in CD. Few small studies, which were summarized in a systematic review, reported about the effect of myopenia and myosteatosis on postoperative course and disease outcome in CD patients. This review showed an association between radiological assessment of myopenia and major postoperative complications [29]. However, there were some essential limitations in the included studies. Firstly, there was a considerable heterogeneity regarding the definition of myopenia across all five studies. Notably, the universal term myopenia was suggested to indicate the presence of clinically relevant muscle wasting due to any illness at any age [19] and should be preferred rather than the term sarcopenia for a clinically relevant degree of muscle depletion, which was defined as the age-associated muscle wasting in the elderly. Additionally, the method of muscle depletion assessment was variable using different parameters without standardized cut-offs. The included studies are based on small cohorts of both ulcerative colitis and CD patients with a high heterogeneity in the surgical procedures using CT-based measurements. Another study analyzing CT scans from 114 CD patients after bowel resection found an increased incidence of major postoperative complications in myopenia patients (2.3% vs. 15.7%, *p* = 0.027). In multivariate analysis, myopenia and a decreased SMI were independent risk factors of major postoperative complications in patients with CD [16].

As most CD patients are rather young and MRI is widely performed for preoperative evaluation of CD, we focused on the assessment of muscle mass and signal intensity measured only in MR images. In the literature, there are only two known smaller scaled studies using different MRI sequences to assess myopenia or myosteatosis in patients with CD. Spooren et al. [24] evaluated T1-weighted fat-saturated post-contrast images in 35 CD patients, while Celentano et al. [25] assessed axial T2-weighted sequences in 31 CD patients. In these studies, myopenia and myosteatosis were associated with a higher rate of postoperative complications and unfavorable disease outcome, indicating potential clinical relevance for these two parameters [24, 25]. In our cohort of 223 CD patients, we investigated the role of MRI-defined myopenia and myosteatosis with anastomotic leakage, postoperative complications, and recurrence rates based on a homogeneous cohort, including only patients with Crohn’s disease undergone ileocecal resection. Moreover, myopenia and myosteatosis were assessed using a standardized approach described by van Dijk et al. [22] by a board-certified radiologist, and patients were included only if MRI scans were in line with high-quality criteria as they described above. However, our study could not confirm the results of the preceding studies. The studies referred above, using either CT or MRI scans to assess myopenia or myosteatosis, included a limited number of participants. Considering that our study includes essentially more patients after performing a power analysis, MRI myopenia and myosteatosis parameters might not be adequate markers to predict postoperative outcome or recurrence rates for CD patients.

Concerning postoperative outcome measured by length of hospital stay, a previous study including 77 IBD patients requiring resection surgery reported that CT-based myosteatosis was significantly associated with increased hospital stay postoperatively (9 versus 13 days) and increased 30-day hospital readmission rates [30]. One-third of the included patients did not suffer from CD but either from ulcerative colitis (27%) or indeterminate colitis (5%). Notably, no multivariate analysis was reported in this study regarding the role of myosteatosis on hospital stay, whereas multivariate analysis was performed for other parameters, and myosteatosis was evaluated using CT scans. In our study analyzing MRI scans of 223 patients, although patients with longer hospital stay had a higher myosteatosis index in the univariate analysis, myosteatosis was not an independent risk factor for longer hospital stay in the multivariate regression analysis.

Chronic inflammation and malnutrition strongly characterize CD patients [31, 32]. Validation of myopenia and sarcopenia definitions that include both low muscle mass and poor muscle function is needed [33]. In the current revised guidelines of European Working Group on Sarcopenia in Older People (EWGSOP2), muscle strength comes to the forefront, as it is recognized that muscle strength may be more accurate than mass in predicting adverse outcomes [34]. Consensus operational definition of sarcopenia includes low muscle strength, low muscle quantity or quality, and low physical performance [34]. In line with the definition of sarcopenia, myopenia assessment should also include muscle function besides muscle mass. However, muscle function quality was not taken under consideration in the available studies on myopenia and myosteatosis concerning surgical CD patients. Easily, standardized muscle function tests are needed to be included in future studies and their results to be combined with muscle mass quantity and quality parameters.

Although the present study is single center based with a retrospective design, our cohort is homogeneous including exclusively CD patients who underwent ileocecal resection and MRI-based assessment of myopenia and myosteatosis. However, due to retrospective study design, not all clinical data were available, e.g., preoperative dose of steroids, interval from infusion of biologicals to surgery, and preoperative serum albumin levels to assess nutritional status of the patients. Furthermore, the present study included only patients with a preoperative available MRI enterography. Thus, about one-third of our patients undergone ileocecal resection for terminal ileitis were excluded from analysis. Still, to our knowledge, this is the largest study exploring the potential association of MRI-defined myopenia and myosteatosis with postoperative complications and recurrence rates after ileocecal resection for Crohn’s disease based on a homogeneous cohort with available high-quality MRI scans.

In conclusion, MRI-based assessment of myopenia and myosteatosis is easily feasible in CD patients. However, we found no evidence in our retrospective study that any of the analyzed MRI muscle quantity or quality parameters are reliable predictors of anastomotic leakage, postoperative complications, or recurrence in CD patients undergoing ileocecal resection. Further studies considering skeletal muscle function besides muscle mass may be needed to further evaluate myopenia and myosteatosis in CD. This may potentially reveal reliable markers for preoperative stratification of CD patients, help to identify high-risk patients, and thus may allow for prevention of unfavorable outcomes.

## Data Availability

The data that support the findings of this study are available from the corresponding author upon reasonable request.
